# Biomarkers for Allogeneic HCT Outcomes

**DOI:** 10.3389/fimmu.2020.00673

**Published:** 2020-04-21

**Authors:** Djamilatou Adom, Courtney Rowan, Titilayo Adeniyan, Jinfeng Yang, Sophie Paczesny

**Affiliations:** ^1^Department of Pediatrics, Indiana University School of Medicine, Indianapolis, IN, United States; ^2^Department of Microbiology and Immunology, Indiana University School of Medicine, Indianapolis, IN, United States; ^3^Melvin and Bren Simon Cancer Center, Indiana University School of Medicine, Indianapolis, IN, United States

**Keywords:** biomarkers, graft-versus-host disease, graft-versus-tumor, hematopoietic cell transplantation, proteomics

## Abstract

Allogeneic hematopoietic cell transplantation (HCT) remains the only curative therapy for many hematological malignant and non-malignant disorders. However, key obstacles to the success of HCT include graft-versus-host disease (GVHD) and disease relapse due to absence of graft-versus-tumor (GVT) effect. Over the last decade, advances in “omics” technologies and systems biology analysis, have allowed for the discovery and validation of blood biomarkers that can be used as diagnostic test and prognostic test (that risk-stratify patients before disease occurrence) for acute and chronic GVHD and recently GVT. There are also predictive biomarkers that categorize patients based on their likely to respond to therapy. Newer mathematical analysis such as machine learning is able to identify different predictors of GVHD using clinical characteristics pre-transplant and possibly in the future combined with other biomarkers. Biomarkers are not only useful to identify patients with higher risk of disease progression, but also help guide treatment decisions and/or provide a basis for specific therapeutic interventions. This review summarizes biomarkers definition, omics technologies, acute, chronic GVHD and GVT biomarkers currently used in clinic or with potential as targets for existing or new drugs focusing on novel published work.

## Introduction

Allogeneic hematopoietic cell transplantation (allo-HCT) remains the most widely used immunotherapy for the treatment of many hematologic disorders. While HCT induces beneficial graft-versus-tumor (GVT), the development of graft-versus-host-disease (GVHD) remains a major cause of mortality and morbidity in patients post-HCT. There are two main clinical presentations of GVHD: acute GVHD (aGVHD) and chronic GVHD (cGVHD). aGVHD affects up to 50% of allo-HCT recipients, and is characterized by an exacerbated inflammatory response and a combination of signs and symptoms that target the skin, liver and the gastrointestinal track. The clinical manifestation of aGVHD includes nausea, vomiting, anorexia, watery or bloody diarrhea with crampy abdominal pain, maculopapular rash, and cholestatic liver disease (1, 2). On the other hand, cGVHD develops in up to 70% of allo-HCT recipients and clinically involves a plethora of organ systems including the oral, musculoskeletal, and genital, and is also similar to immune diseases such as scleroderma. cGVHD is the most long-lasting complication of allo-HCT and results in high non-relapse mortality (NRM) in up to 12% of cases, organ dysfunction, high morbidity, and impaired quality of life (3–5). While HCT with HLA-matched unrelated donor, HCT with HLA-mismatch related donor, older recipient and donor age, the use of female donor for male recipients are all risks factors associated with the development of cGVHD, high grade aGVHD is associated with an increased risk of cGVHD development in patients (6). Unfortunately, patients at high risk of treatment unresponsiveness, GVHD morbidity, or even death fail an early diagnosis due to the lack of early prognostic tools that would enable to identification of patients before disease onset.

Over the years, advances in bioinformatics including machine learning, chemistry, engineering, and high-throughput technical instruments have massively contributed to the development of “omics” technologies. Using these tools, several novel specific and sensitive blood based-biomarkers were identified and validated in large patient’s cohort to aid in the diagnosis, prognosis, risk prediction, and response to therapy of patients post-HCT. These biomarkers can serve as potential therapeutic targets for existing or novel drugs and also be exploited to facilitate with the diagnosis and clinical assessment of disease severity in patients to enable an optimal clinical management during disease progression.

This review will summarize these novel drug-targetable aGVHD, cGVHD and GVT biomarkers post-HCT identified using a large number of patients (cutoff of at 50 patients per cohort), a validation cohort, and validated at the protein level with the potential for rapid translation into the clinic.

## Biomarkers Definitions

The Working group on biomarkers for the National Institutes of Health Consensus Development Project on Criteria for Clinical Trials in Chronic Graft-versus-host disease (GVHD) report and the North American and European Consortium put forward a list of definitions for GVHD biomarkers (7, 8). A biomarker mostly refers to a biochemical variable, such as a circulating protein or a biomolecule and is categorized into four major definitions:

(1) *A diagnostic biomarker* is used to identify GVHD patients at the onset of the disease and aid to differentiate their symptoms from other conditions.
(2) *A prognostic biomarker* is used to identify patients with different degree of risk for GVHD occurrence, progression or resolution before the onset the disease.
(3) *A predictive biomarker* categorizes patients based on their likelihood to respond to therapy before GVHD therapy.
(4) *A response to treatment* biomarker aids monitor patients’ response to treatment when pre-therapy sample is collected.

## Biomarker Development Phases

The development of biomarkers is complex and consists of multiple phases, from candidate molecular targets to routine use in the clinics. Prior to prospective studies, validation with both training and verification cohorts, then validation in independent cohorts must be conducted (7, 8). The different phases are detailed below:

### Discovery Phase

First, using a discovery phase small scale cohort of 20 to 40 cases and controls are compared using tools mentioned in the next paragraph. Statistical analysis to evaluate the accuracy of biomarkers relies on the AUC of ROC, which is one the most objective biomarker performance evaluation. It measures specificity on the *x*-axis versus 1 minus sensitivity on the *y*-axis for every possible cut off (9). A biomarker can be evaluated using the following guidelines: AUC of 0.9–1.0 = excellent; AUC of 0.8–0.9 = good; AUC of 0.7–0.8 = fair; AUC of 0.6–0.7 = poor; and AUC of 0.5–0.6 = fail (9). Candidate biomarkers with enough specificity and sensitivity determined by an area under the curve (AUC) of the receiver operating characteristic (ROC) >0.70, will move forward to the next phase of qualification.

### Qualification Phase

In the second phase, the few biomarkers that were selected in the discovery phase are now evaluated with a qualified assay. An assay is qualified using important analytical parameters such as the specificity, accuracy, precision, robustness, limit of quantitation, linearity, range, ruggedness and detection limit. It is important to note that the finalized assay cannot be changed without requalification of the assay under the revised conditions.

### Validation Phase

The last phase will lead to the biomarker able to be used in a clinical trial to test its impact on patient outcomes using the qualified assay as described above (7, 8).

## Tools Used in the Identification of Biomarkers

First, a note on samples to be collected, which should ideally be non-invasive, and allowing for multiple time points collection. Therefore, biofluids such as sera, plasma, and urine are highly preferred. Furthermore, most repositories contain plasma and sera because they are easy to process and store. Another non-invasive sample is urine, but its protein composition is inherently biased by renal filtration.

Over the last decades, advances in omics technologies have allowed for the analysis of a broad spectrum of molecular changes in a single cell or an organism to provide information regarding a disease. Omics is defined as the complete sets of molecules, including proteomics, cytomics, transcriptomics, and genomics that were facilitated by engineering and provided increased data throughput (10, 11). In the next section, the different omics technologies used for the identification of biomarkers will be discussed.

### Profiling Using Genomics

Patients’ outcomes post-allo-HCT can be improved by strategies that aim at (1) reducing peri-transplantation risk and (2) facilitating diagnosis and prognosis of HCT-related complications. Gene signatures were previously associated with GVHD prevention and management. Single chain polymorphism (SNPs) are the most common types of naturally occurring mutations in a population. In a retrospective study, a total of 25 SNPs in 12 cytokine genes were evaluated in a cohort of 509 HLA-identical sibling donor allo-HCT patients for the prediction of aGVHD and cGVHD. Using a linear regression model and the least absolute shrinkage and selection operator (LASSO), SNPs combined with other clinical factors could predict severe GVHD (12). Recently, a genome-wide association studies (GWASs) of polymorphism showed that although the number of minor HLA mismatches was double in non-related transplants compared to sibling HLA-matched transplants, GVHD outcomes were higher in HLA-DP GVHD-mismatched unrelated recipients than in HLA-matched related recipients, demonstrating that increased GVHD development after unrelated-HCT is mostly due to HLA-mismatching (13). Another GWAS study of 3,532 patients, known as the Discovery-BMT study demonstrated the association with SNPs in the major histocompatibility complex II and overall survival post HLA-matched unrelated donor HCT (14). Functional single nucleotide polymorphisms in the major histocompatibility complex II are associated with overall survival after HLA matched unrelated donor BMT). Unfortunately, large patients’ cohorts investigating candidate-genetic polymorphism were unable to confirm findings from a previous smaller cohort for both aGVHD and cGVHD indicating that most published SNPs are not reproducible because they were either non-functional or missing important functional genetic elements (15, 16). However, in a more recent study, donors SNPs of IL1RL1 exhibited a strong correlation with pre-transplantation serum/plasma levels of Stimulation-2 (ST2), which is also known as IL-33 receptor, and an association with the risk of aGVHD and potential donor selection implication (17).

### Profiling Using Proteomics

Due to the complexity of data analysis and data acquisition, the use of proteomics analysis is mostly limited to specialized laboratories. Yet, the main advantage is that biomarkers discovered through proteomics actually indicate the state of the disease. GVHD biomarkers have been discovered using proteomics analysis. Antibody arrays are quantitative and highly sensitive for the detection of low-abundance proteins such as cytokines. Their main limitation is the restricted number of antibodies on the array, thus affecting the discovery of candidate biomarkers. Another powerful tool for qualitative and quantitative discovery of proteins in a complex protein mixture is next generation mass spectrometry (MS), which uses a gel-free separation method for the first step most likely liquid chromatography, followed by MS. MS, particularly tandem MS uses label-free methods or isotopically labeled tags for non-ambiguous quantification. Proteins are identified from a mass spectra matched to a sequence database (18). Although these methods are the most efficient for biomarker discovery in clinical research, these approaches are too time consuming to use in validation.

Despite the great promise for biomarker discovery using next-generation MS, they are limitations between biomarker validation and discovery due to (1) the paucity of affinity-capture reagents that has led to bias in the prioritization of candidate biomarkers, and (2) the increase in the number of samples necessary for validation that augments when a biomarker passes to each test phase, thus creating the need for high-throughput assays. Sandwich enzyme linked immunosorbent assay (ELISA) is the most specific and reliable approach for the quantification of individual proteins because this method is simple, very easy to perform with high reproducibly (3).

### Profiling Using Cytomics

Flow cytometry and mass cytometry are high-throughput methods used for the profiling of immune cell populations. CYTOF is a time-of-flight MS approach used for the measurement of several markers on cells. This approach is similar to flow cytometry, except for the use of heavy metals ion tags labeled antibodies instead of fluorochromes. The main advantage of CYTOF over flow cytometry is that more antibody specificities can be used in a single sample (classically 30–40 antibodies), without significant spillover between channels. Although CYTOF is limited to the markers used, this technology and its software have enabled the discovery of new cell populations such as regulatory T cells (Tregs) (19–22), B cells (23, 24), T follicular helper (TFH) cells (25), T follicular regulatory (TFR) (26) cells, and invariant natural killer T cells (27), which will be discussed below. In addition, proteomics with flow cytometry or mass cytometry enabled the discovery of a new subset of T cells including the CD4^+^CD146^+^CCR5^+^ T cells in aGVHD or cGVHD, and the blood mucosal-associated T cells (CD161^+^TCRVα7.2^+^ and CD38^+^ T cells in cGVHD (28–30). Although these set of immune cells provide great insight into the pathophysiology of GVHD and are good therapeutic targets, they remain less ideal biomarkers than soluble molecules measurable by ELISA, due to the relatively low throughput associated with cytomics, the lack of standard curve for quantification, and the need for large samples of fresh blood. However, they remain best markers of response to a specific treatment (e.g., Tregs, TFH cells, and TFR cells post-IL2 therapy) (22, 26).

### Profiling Using Transcriptomics

Transcriptomics refers to an organism’s transcriptome, or the sum of all its RNA transcripts, including mRNAs, ln RNAs and small RNAs (31). Studies of gene signatures of GVHD can be classified as candidate-gene studies and genome-wide studies, and also offer less bias in the identification of genes, pathways, and gene expression networks active in the disease (3). In the last years, transcriptomics analysis has led to major discoveries in the fields of infectious disease, vaccinology, and solid organ transplantation. Transcription analysis is mostly performed using bulk peripheral blood mononuclear cells (PBMCs), rather than whole-blood approaches as it limits contamination by granulocytes. Although not independently validated in a cohort, a classifier of 20 genes was discovered in allo-HCT patients, and differentiates tolerant vs. non-tolerant patients (32). In another multicenter study conducted by Chronic Disease Consortium, an identifier of 3 different RNA biomarkers genes, IRS2, PLEKHF1, and IL1R2, and two variables (recipient cytomegalovirus serostatus and conditioning regimen intensity) accurately identified cGVHD patients from controls (AUC = 0.81) (33). Although total mononuclear cells can be utilized for transcriptomics and the identification of biomarkers; this approach is not accurate as the largest cell population, which is not reflective of the pathogenic cells, will dominate. Therefore, specific subset of immune cell population is sometimes used for RNA isolation. For instance, T cells, which are associated with the pathogenicity of GVHD have been sorted, then used for RNA isolation. Other novel identified drivers of GVHD included programmed death ligand 1 (PD-L1) on donor T cells, proinflammatory cytotoxic T cell 17 (Tc17), and several other miRNAs (34–37). Using the highly translational non-human primate (NHP) model, another group studied the transcriptional signatures of T cells during breakthrough aGVHD and hyperacute GVHD (38). They used sorted CD3^+^ T cells in NHP and CD4^+^ and CD8^+^ T cells in humans in both supervised and unsupervised gene expression analyses for the identification of pathways controlling GVHD, and discovered three transcriptional hallmarks of breakthrough aGVHD that are not observed in hyperacute GVHD: (1) T cell persistence rather than proliferation, (2) a highly inflammatory programming, (3) a T helper (Th)/Tc1-mediated dysfunction driven by inflammatory IL-17 dominated pathways (38). They further demonstrated the role of Aurora Kinase A and the OX40:OX40L pathways as novel mediators of aGVHD induced in both the NHP and human alloreactive T cells that can be blocked with the combination of mammalian target of rapamycin inhibition with sirolimus to induce long-term control of both hyperacute and breakthrough aGVHD (39, 40). More recently, monoprophylaxis with FR104, an antagonistic CD28-specific pegylated-Fab’, or the combined prophylaxis with sirolomus/FR104 enhanced the control of effector T cell activation and proliferation to control GVHD in NHPs (41). In circulating monocytes in cGVHD compared to monocytes from normal subjects and non-cGVHD, two pathways were upregulated: (1) interferon (IFN) inducible genes (MX1, CXCL9, CXCL10) and innate receptors for cellular damage (Toll-like receptor 7 and DDX58) (42).

#### Metabolic Biomarkers in GVHD

More recently, another study performed both global metabolic analysis and transcriptomic profiling in two separate cohorts of allo-HSCT recipients with or without aGVHD in order to detect novel aGVHD biomarkers. Pathway analysis of 38 altered metabolites and 1,148 differentially expressed gene surrogates revealed a distinct glycerophospholipid metabolism signature of aGVHD with predictive value (43). Although both a discovery and validation cohort of 50 and 70 patients, respectively, were used, this study has few limitations as (1) it has a relatively low number of patients in each set that were selected to be positive or negative not representing an all-comers population, and (2) the biomarker validation at the protein level can more rapidly be translated into a test for clinical application.

### Analytical Tools

Beyond the classical statistics reviewed elsewhere (44), machine learning methods are artificial intelligence tools stemming from computer sciences that are used to learn information directly from data without relying on a predetermined equation as a model (45). One of the main advantages of this approach is that it can process large amounts of data. In a retrospective study of 28,236 HCT-patients from the European Blood and Marrow Transplantation (EBMT) registry, 10/20 variables were selected by the alternating decision tree (ADTree) model for overall mortality at 100 days post-HCT which performed better than the classical EBMT score (AUC of 0.701 vs. 0.646, *p* < 0.001) (46). Using the same algorithm, they confirmed this finding in a smaller cohort of 1,848 patients from the Italian Transplantation registry (GITMO) (AUC of 0.698 for day 100 mortality) (47). Furthermore, a recent study from the Japanese Transplant Registry asked with similar method (ADTree) if they would predict aGVHD grade II-IV in a cohort of 26,695 HCT patients. Using 15/40 variables, they predicted aGVHD grade II-IV with an AUC of 0.616. The authors went on to validate these 15 variables with conventional statistics and showed a cumulative incidence of aGVHD II-IV of 58.9% with the high-risk score and 29% in the low risk score (48). This type of method can also be used at a smaller scale to identify new features in complex phenotypes such as cGVHD. For example, in one study, the authors compared cause-specific hazard function to the Bayesian Additive Regression Tree (BART) model in a cohort of 845 patients with 427 cGVHD, and showed that BART performed as well as cause-specific hazard function (49). Another study with 339 patients with cGVHD features, revealed that patients in the high- and intermediate-risk decision-tree groups had significantly shorter survival than those in the low-risk group (hazard ratio 2.74; 95% confidence interval: 1.58–4.91 and hazard ratio 1.78; 95% confidence interval: 1.06–3.01, respectively) (50). More recently, another study used machine learning to assess the effects of immune parameters on clinical outcomes after HLA-haploidentical and HLA-matched allogeneic bone marrow transplantation with posttransplant cyclophosphamide (PTCy). Findings showed that (1) NK cell recovery can predict survival after both HLA-haploidentical and HLA-matched HCT with PTCy, (2) early CD4^+^ T-cell recovery and higher CXCL9 levels can predict development of acute GVHD, and (3) high Reg3α levels at day 56 predict the development of chronic GVHD, demonstrating that machine learning can be utilized to demonstrate the association of immune cell subsets and biomarkers with outcomes after HCT (51). Machine learning has several strengths: (1) the model handles a number of complexities in modeling, including interactions, high-dimensional parameters. However, there are two main weaknesses: (1) at the exception of tree algorithms, it is not straightforward for the clinicians to directly interpret the models by themselves (black box) and (2) it requires a large sample size to train the model.

## Validated Biomarkers Post-HCT

Over the years, several biomarkers have been discovered and validated in both aGVHD and cGVHD. According to the NIH consensus on biomarkers, some proteins were moved from candidate proteins to biomarkers (7). Those validated biomarkers will be discussed in the section below and summarized in Table 1.

**TABLE 1:** Plasma and cellular biomarkers for post-HCT outcomes.

**Protein**	**Study**	**No. of patients in the study**	**Association direction**	**Diagnosis timepoint (median day post-HCT)**	**Prognostic timepoint (median day post-HCT)**	**References**
**Plasma aGVHD**						
4 biomarker panel: IL-2-receptor-α, hepatocyte growth factor (HGF), IL-8 tumor necrosis factor receptor-1	Paczesny 2009	42+282^†^+142^†^	Increased	28	ND	52
Interleukin-6 (IL-6)	McDonald 2015	74^†^+76 ^†^	Increased	28	Not significant	53
	Kennedy 2014	53	Increased (3–14), then decreased	30	7–14	54
Stimulation 2 (ST2)	Vander Lugt 2013	20+381^†^+673*+75*	Increased	28	14	55
	Levine 2015	328+164^†^+300^†^	Increased	28	ND	56
	Abu Zaid 2017	211 patients (independent cohort post-validation)	Increased	28	ND	58
	McDonald 2015	74^†^+76^†^	Increased	28	Not significant	53
	Hartwell 2017	620+309^†^+358^†^	Increased	ND	7	59
	Major-Monfried 2018	236+142^†^129^†^	Increased	ND	7	64
	McDonald 2017	165	Increased	ND	14	65
T cell immunoglobulin domain and mucin domain (TIM3)	McDonald 2015	74^†^+76^†^+167*	Increased	28	14	53
	Abu Zaid 2017	211 patients (independent cohort post-validation)	Increased	28	ND	58
AREG/EGF ratio	Holtan 2016	105+50^†^	Increased	160	ND	66
**Skin specific**						
Elafin	Paczesny 2010	522+492^†^	Increased	28	ND	67
	Bruggen 2015	59	Increased	28	ND	68
**Liver specific**						
REG3 α, HGF, and Keratin 18 (KRT18)	Harris 2012	954, 3 centers	Increased	14	28	69
**GI specific**						
Regenerating islet-derived 3-α (REG3 α)	Ferrara 2011	20+871^†^143^†^	Increased	28	ND	71
T cell immunoglobulin domain and mucin domain (TIM3)	Hansen 2013	20+127^†^+22^†^	Increased	28	ND	73
**Cellular aGVHD**						
Regulatory T cells	Magenau 2010	215	Decreased	3–14	28	19
CD146+ T cells	Li 2016	20+214^†^	Increased	ND	14	28
CD30	Chen 2012	53	Increased	ND	ND	80
	Chen 2017	34	Increased	ND	NA	81
Invariant natural killer T cells (iNKT)	Chaidos 2012	57	Increased	ND	NA	82
**Plasma chronic GVHD**						
sBAFF	Sarantopoulos 2007	104	Increased	480	NA	93
	Fujii 2008	80	Increased	171 (early^&^), 429 (late^&^)	NA	94
	Kitko 2014	35+109^†^+211^†^	Increased, and not validated in independent cohort	154^+^, 256 (early^&^), 619 (late^&^)	NA	95
	Kariminia 2016	23+198^†^+83^†^	Increased	203,174	NA	96
	Saliba 2017	341	Increased/decreased #	189	NA	98
CXCL9	Kitko 2014	35+109^†^+211^†^	Increased	154^+^, 256 (early^&^), 619 (late^&^)	NA	95
	Yu 2016	53+211^†^+180^†^	Increased	210^+^,203^&^	100	99
	Kariminia 2016	23+198^†^+83^†^	Increased, and not validated in independent cohort	203^+^,174^&^	NA	96
	Hakim 2016	26+83^†^	Increased	132	NA	42
	Abu 2017	211	Increased	NA	100, 180, 365 (time dependent analysis)	58
CXCL10	Kariminia 2016	23+198^†^+83^†^	Increased	203^+^,174^&^	NA	96
	Hakim 2016	26+83^†^	Increased	132	NA	42
	Ahmed 2016	78+37	Increased	132	NA	97
Four protein panel (CXCL9, ST2, OPN, MMP3)	Yu 2016	53+211^†^+180^†^	Increased	210,203	100	99
MMP3	Liu 2016	76 (BOS)	Increased	531	NA	100
CCL15	Du 2018	211^†^+792^†^	Increased at onset, but not prognostic	203	100	101
**Cellular chronic GVHD**						
CD163	Inamoto 2017	40+127^†^	Increased	NA	80	102
B cells						
TLR9^+^	She 2007	54	Increased	171 (early), 429 (late)	NA	103
CD21^*low*^	Greinix 2008	70	Increased	1428	NA	104
	Kuzmina 2013	136	Increased	143	NA	105
BAFF/B cell ratio	Sarantopoulos 2009	57	Increased	180	NA	23
Tregs	Zorn 2005	57	Decreased	720	NA	20
CD4^+^CD146^+^CCR5^+^	Forcade 2017	40	Increased	942	NA	29
TFH	Forcade 2016	66	Decreased	867	NA	25

*ND, not done; NA, not applicable; ^†^Patient number in validation cohort 1 and cohort 2; ^+^cohort 1 and ^&^cohort 2.*

### Acute GVHD Biomarkers

#### Plasma Biomarkers

##### Systemic biomarkers

###### A panel of 4 biomarkers: IL-2 receptor-α (IL-2Rα), tumor necrosis factor receptor-1 (TNFR-1), IL-8, and hepatocyte growth factor (HGF)

Screening of aGVHD plasma samples using antibody microarrays for 120 proteins and ELISA enabled for the discovery and validation of the first panel of biomarkers consisting of a 4-protein biomarker panel: IL-2Rα, TNFR-1, IL-8, and HGF. This panel of biomarkers can confirm the diagnosis of aGVHD in patients, and COX regression analysis revealed that the panel can also predict survival independent of GVHD severity (52).

###### Interleukin-6 (IL-6)

Interleukin-6 was identified as a predictive biomarker for severe GVHD and NRM at both days 3 and 60 post-transplant in a cohort of 53 HCT patients. This finding was then validated in a second cohort, where IL-6 was elevated at the onset of GVHD (53). In a subsequent study, blockade of IL-6 using tocilizumab in addition to standard GVHD prophylaxis reduced the incidence of aGVHD (54).

###### Stimulation-2 (ST2)

Stimulation-2, the IL-33 receptor or IL1RL1 gene product remains the most validated biomarker for aGVHD and non-relapse mortality (NRM) either measured alone or with other markers. (1) ST2 serves as a predictive biomarker. ST2 was first identified in plasma obtained at a median of 16 days after the initiation of aGVHD therapy in 10 patients with a complete response by day 28 post-therapy initiation and compared to 10 patients with progressive aGVHD during therapy. In that study, 12 biomarkers were compared, and ST2 showed the highest association with resistance to aGVHD and death without relapse. Patients with high ST2 levels had a higher risk to develop treatment resistant-aGVHD compared to patients with low ST2 levels (55). Additionally, ST2 could predict the development of aGVHD independent of aGVHD grade (55). ST2 was subsequently validated as a predictive biomarker in a larger cohort of 492 HCT patients with newly diagnosed GVHD. High ST2-based GVHD scores were associated with a lower response rate to aGVHD treatment (56). Of note, the authors called this a prognostic score when it was a predictive score. ST2 has since been tested in a multi-center, open-label, randomized clinical trial conducted by the Blood and Marrow Transplant Clinical Trials Network (Study 1501, NCT02806947). This study evaluated the difference in day 28 complete response (CR) and partial response (PR) to sirolimus (steroid-free regimen) as compared to prednisone as an initial treatment for patients with Minnesota standard-risk and low-risk biomarker-confirmed aGVHD. This study showed no difference in day 28 CR/PR rates for sirolimus 64.8% (90% Cl 54, 1%–75.5%) compared to 73% (90% Cl 63.8%–82.2%) for prednisone (57). This shows that biomarker can aid clinicians opt for a lesser toxic aGVHD regimen. (2) ST2 as a prognostic marker. ST2 levels in patients at day 14 post-HCT, prior to any clinical manifestation of aGVHD, were associated with 6-month NRM (55). Similar findings were made in several other studies including a phase 3 multicenter study of 211 patients where high ST2 at day 28 post-HCT were associated with 2 year- NRM (58). In another study, a biomarker algorithm based on ST2 plasma levels collected at day 7 post-HCT could consistently predict the 6-month NRM in high risk (28%) vs. low risk patients (7%), *p* < 0.001 (59). In a third confirmatory study, plasma ST2 levels were also prognostic for the development of aGVHD (53). We note that in this study the authors use the term predictive instead of the recommended prognostic term. Furthermore, the prognostic value of ST2 has been shown in patients cohorts receiving other HCT platforms such as HCT with non-myeloablative conditioning regimen (60), cord blood HCT (single or double) (61), HCT post cyclophosphamide as aGVHD prophylaxis (62). In a contemporary multicenter center cohort of 415 patients (170 children ≤10 and 245 subjects >10 years (both children and adults) recently published, landmark analyses showed for the first time that pre-HCT high ST2 was significantly associated with NRM particularly in children age ≤10 years [HR (CI): 4.82 (1.89–14.66), *p* = 0.0056 (63). (3) Last, ST2 as a response to treatment marker. High ST2 and Regenerating islet-derived 3-α (REG3α) when monitored as early as 1 week after the initiation of treatment determined the non-responder rates (64). Similar findings were reported with the combination of ST2 and T-cell immunoglobulin mucin-3 (TIM3) at 14 days post initiation of prednisone (64, 65).

###### Amphiregulin (AREG)-to-epithelial growth factor (EGF) ratio

The role of angiogenic factors in late aGVHD was tested by comparing controls and cases aGVHD patients in a cohort of 105 patients, then validated in a cohort of 37 cases. The authors found that AREG-to-EGF ratio at or above median was associated with lower overall survival and higher NRM in both cohorts. AREG-to-EGF ratio was also elevated in classic aGVHD, but not in cGVHD (66). This finding was not validated in an independent cohort. However, the study showed that patients with aGVHD and high AREG (≥33 pg/ml) had a lower response rate to steroid, higher NRM, and lower overall survival (66).

##### Organ-specific biomarkers

Certain biomarkers are organ specific and enable the distinction for instance from skin rashes and skin GVHD, or other forms of enteritis to GI-GVHD. Target-specific aGVHD biomarkers are:

###### Skin specific Elafin

Elafin was also discovered using next-generation proteomics and validated as both a diagnostic and prognostic biomarker for skin GVHD, which is associated with GVHD severity and NRM (67, 68).

###### Liver specific REG3α, HGF and cytokeratin-18-fragments (KRT18)

Regenerating islet-derived 3-α, HGF, and KRT18 were elevated in patients with liver GVHD in a cohort of 954 patients from three centers. It is important to note that REG3α had a better AUC for the diagnosis of liver GVHD than HGF and KRT18. However, this panel of liver GVHD specific biomarker was not validated due to the low incidence of liver GVHD (69, 70).

###### GI-Specific- Regenerating islet-derived 3-α (Reg3α) and T-cell immunoglobulin mucin-3 (TIM3)

T-cell immunoglobulin mucin-3 and Reg3α are GI-GVHD specific biomarkers that were identified and validated as prognostic biomarkers that can identify patients at high risk for lethal aGVHD at day 7 and day 14 for each, respectively (53, 59). Using next generation proteomics, Reg3α and TIM3 were discovered at higher levels in the lower GI of aGVHD. This finding was subsequently validated in multiple cohorts either alone or in combination with other markers (53, 69, 71–73).

#### Cellular Biomarkers

##### Regulatory T cells (Tregs)

CD4^+^CD25^hi^Foxp3^+^ Tregs showed both a diagnostic and predictive value as a biomarker for aGVHD. Lower Tregs in the peripheral blood of patients was associated with the development of aGVHD. Furthermore, patients with Tregs frequencies lower than the median exhibited higher NRM compared to patients with Tregs higher than the median (19). This finding was confirmed in another study where Tregs suppressed the proliferative effects of conventional T cells, and promoted a significant protection from lethal aGVHD (74). Furthermore, Tregs were able to suppress the early expansion of alloreactive donor cells, their IL-2R expression and their capacity to induce aGVHD (75). One relevant study showed that the infusion of *ex vivo* activated and expanded Tregs inhibited aGVHD lethality (76). A more recent study showed that daily therapy with low levels of interleukin-2 (IL-2) in patients improved CD4 Tregs-suppressive function, and that better patients clinical response seen with low dose IL-2 therapy was associated with an increased diversity of the CD4 Tregs TCR repertoire in patients with cGVHD (77). Please refer to the recent comprehensive review that analyzes the role of T regs in both cGVHD and aGVHD (78).

##### CD4^+^CD146^+^CCR5^+^ T cells

Using in-depth, large-scale proteomic profiling of presymptomatic samples, a T cell population expressing CD146, an adhesion molecule, was found upregulated as early as 14 days post-transplantation in patients with increased risk of GI-GVHD. This population of T cells was also induced by ICOS stimulation. shRNA knockdown of CD146^+^ in T cells reduced the infiltration of pathogenic TH17 cells to the gut, and increased their survival and the frequency of Tregs (28).

##### CD30

Although not validated, CD30, a cell-surface protein found on certain activated T cells, was highly expressed on the CD8^+^ T cells or the plasma of aGVHD patients (79). In a subsequent multicenter phase 1 clinical, brentuximab, an antibody-drug conjugate targeting CD30, showed 38% response rate in steroid-refractory GI-aGVHD patients (80).

##### Invariant natural killer T cells (iNKT cells)

High levels of iNKT cells in donor graft were associated with a decrease in GVHD development. Patients that received CD4^–^iNKT-cell doses above the median had a cumulative incidence of grade II-IV of 24.2% compared to 71.4% in patients with low iNKT-cell dose, *p* = 0.0008. This finding was also not validated. The same finding was found in mixed lymphocyte reaction assays where CD4^–^iNKT T cell suppressed T cell proliferation and IFNγ secretion in a contact-dependent manner (81).

##### Microbiota as a biomarker in GVHD

In early 1970s, studies in mice provided the first indication that the intestinal microbiota affects the development of GVHD when mice treated with antibiotics or germ-free mice showed a prolonged survival post-allo HCT (82, 83). Then, the use of high-throughput sequencing technologies provided further information on the relevance of the microbiota in GVHD, and specific information on the bacteria that might be detrimental or beneficial post- HCT. The normal human microbiota encompasses different anaerobic commensal bacteria, mostly members of the *Firmicutes* and *Bacteroidetes* phyla (84–86). However, during HCT, a dysbiosis or changes in the microbiota are recorded. In two different studies, an increase in *Enterococcus* and γ*-Proteobacteria*, including *Enterobacteriaceae* were all associated with the development of GVHD and increased mortality in patients post-transplantation (87, 88). On the other hand, increased *Bacteroides* and Clostridium genus *Blautia* were associated with lower GVHD in patients post-HCT. Another study confirmed the loss of microbiota that occurs in the gut post-HCT and found an increase in *Lactobacillales* and a decrease in *Clostridiales* (89). Fecal metabolites can also provide insightful information on GVHD patients’ outcome. For instance, the presence of fecal butyrate and indole, in patients post-HCT directly correlated with enrichment of *Clostridiales* and *Bacteriodales*, respectively, in an analysis of 451 fecal specimen from 44 patients before HCT through 100 days post-HCT. Although fecal butyrate and indole did not impact aGVHD incidence or overall survival in these patients, low levels of butyrate were found in patients contracting blood stream infections within 30 days (90).

### Chronic GVHD Biomarkers

Chronic GVHD is a long-term complication that develops in patients post blood or bone marrow transplantation characterized by autoimmune disease-like symptoms such as scleroderma and Sjogren syndrome. The clinical symptoms of cGVHD include fibrosis and inflammation that affects multiple organs and tissues within the body, thus making the diagnosis of the disease challenging in patients (7, 9). Therefore, validated cellular and plasma biomarkers would be beneficial for the diagnosis, risk stratification and response to treatment in patients post-HCT. Validated cGVHD plasma and cellular biomarkers are listed in the next section.

#### Plasma Biomarkers

##### Soluble B-Cell activating factor (sBAFF)

Different studies demonstrated the role of sBAFF as both a diagnostic and prognostic biomarker in chronic GVHD. High sBAFF levels in patients were associated with active cGVHD and both the early onset of GVHD (3–8 months) as well as late cGVHD (≥9 months) (91–95). sBAFF can also predict response to treatment as greater than a 50% decrease in sBAFF was recorded in responders to corticosteroids at 2 months after the initiation of therapy (92). In a more recent study, increased sBAFF at the time of diagnosis were associated with NRM (96).

##### A panel of 4 biomarkers: ST2, CXCL9, Matrix metalloproteinase 3 (MMP-3) and Osteopontin

A biomarker panel consisting of ST2, CXCL9, MMP-3, and osteopontin was significantly correlated with cGVHD. Furthermore, when measured at diagnosis or at day + 100 post-transplantation, this panel allowed for patient risk stratification according to cGVHD risk (97). MMP-3 was also associated with the development of bronchiolitis obliterans (98). CXCL9 is an interferon-γ-inducible ligand for chemokine (C-X-C motif) receptor 3 (CXCR3), which is expressed on effector CD4 Th1 cells and CD8 cytotoxic T lymphocytes. Several studies showed the upregulation of CXCL9 in cGVHD patients and correlation with GVHD severity (42, 58, 93, 94, 97). Similarly, CXCL10, an inflammatory chemokine that also binds to CXCR3 and is involved with the activation, and recruitment of T cells, NK cells, eosinophils, and monocytes, was also shown to be elevated in cGVHD patients (42, 94). Recently, both CXCL9 and CXCL10 were elevated in cGVHD diagnosis in the first replication cohort, but only CXCL10 in the second (94). In a different study, the upregulation of both CXCL9 and CXCL10 in cGVHD was confirmed using ELISAs (42), and therefore the importance of CXCL9 and CXCL10 in the diagnosis of cGVHD needs to further be evaluated.

##### CCL15

Using a tandem mass spectrometry proteomics analysis using a multiorgan cGVHD model, CCL15, the human homolog of CCL9, was identified as a novel cGVHD biomarker in a cohort of 211 patients. In addition, patients with higher than median levels of CCL15 showed a higher risk of NRM, demonstrating that biomarkers identified through murine proteomics can also enable for the discovery of novel biomarkers in patients (99).

##### CD163

CD163 is a macrophage scavenger receptor that is elevated during oxidative stress. High plasma concentrations of CD163 have been associated with the *de novo* onset of cGVHD. Patients with plasma soluble CD163 concentration at day 80 had a cumulative incidence of *de novo*-onset of cGVHD of 75% vs. 40%, *p* = 0.018, in patients with lower concentration of CD163 (100).

#### Cellular Biomarkers

##### B cells

Toll-like receptor 9 (TLR9) expressing B cells have been associated with the development of cGVHD in patients post-transplantation (101). Immature B cells, defined as CD19^+^/CD21^–^ cells in patients has also been associated with the development of cGVHD (102, 103). Last, high plasma levels of BAFF/B cell ratio was found in cGVHD patients compared to healthy patients (23).

##### Tregs

Tregs were significantly reduced in cGVHD patients, where they are essential for tolerance in cGVHD post-transplantation. In one study, Tregs were evaluated in 57 patients post-HCT, and findings showed that a decrease in CD4^+^CD25^+^T cells in patients with cGVHD compared to patients without cGVHD (*p* = 0.009) (20). In another study, an increase Th17/Treg ratio resulted in chronic liver GVHD (104).

##### CD4^+^CD146^+^CCR5^+^ T cells

A novel subset of CD4^+^ CD146^+^ CCR5^+^ T cells, a TH-17 prone subset of CD4^+^ T cells was highly expressed in cGVHD patients and sensitive to pharmacological inhibition. In a murine model, donor T cells obtained from CD146-deficient mice had significantly reduced pulmonary cGVHD compared to the wild-type mice. Moreover, the CD146-deficient mice had significantly lower pulmonary macrophage infiltration and T cell CCR5, IL-17, and IFN-γ coexpression (29).

##### T follicular helper cells (TFH)

Lower circulating TFH (cTFH) cells have been found in patients with active cGVHD compared to patients without cGHVD. Findings also demonstrated that cTFH are activated and exhibit a Th2/Th17 phenotype that promotes B-cell help function during cGVHD (25).

### Graft-Versus-Tumor (GVT) Biomarkers

In tumor immunotherapy, allo-HCT with donor lymphocyte injection (DLI) promotes tumor cell killing through the GVT effect. However, often times, the GVT effect is limited by the development of aGVHD. Therefore, plasma biomarkers that can distinguish GVT without GVHD would be beneficial. Recently, plasma proteomics and systems biology analyses were conducted on patients who experienced GVT and aGVHD compared to the proteome of patients who experienced GVT without aGVHD. The authors identified a total of 76 proteins that were associated with GVT without GVHD. Additionally, an unique 61-protein signature was also identified in patients with GVT without GVHD. 43 genes of the 61 genes in the protein signature were further confirmed using single-cell RNA sequencing analysis. More importantly, few potential GVT biomarkers such as RPL23, ILF2, CD58, and CRTAM were identified in GVT without GVHD (105). These GVHD-free GVT biomarkers warrant further analysis and validation in other cohorts.

## Pathogenic and Druggable Biomarkers

Biomarkers than can provide insight in the pathogenesis of a disease are even more relevant. For instance, during aGVHD, intestinal stromal cells and intestinal T cells, producers of IFNγ and IL-17, are both sources of sST2, a decoy for IL33. This limits the availability of IL33 to cytoprotective T cells that express the transmembrane form of ST2, which consist mostly of T helper 2 (Th2) cells and ST2^+^ Tregs (106). REG3α is a similar biomarker that can prevent crypt apoptosis and aGVHD (107).

Another important characteristic of a biomarker is its ability to be targeted with therapeutic drugs. In rheumatologic diseases, cytokines have been identified as markers and targeted with Janus Kinase (JAK) inhibitors either directly or via intracellular signaling (108). This is also the case in cancer, where signal transducer and activation of transcription 3 (STAT3) is a great potential therapeutic candidate that has been targeted using a small-molecule degrader for complete tumor regression (109). Similar drug targetable biomarkers in GVHD would be very beneficial as these would target the specific biomarker to reduce GVHD, promote therapy, and lower toxicity. In aGVHD, peritransplantation blockade of sST2 using a neutralizing monoclonal antibody or small molecule inhibitors in a murine aGVHD model significantly reduces disease severity and mortality, as well at increase plasma levels of IL-33, lower the donor T cell infiltration to the gut, and IFNγ-producing T cells, while increasing cytoprotective ST2 expressing T cells (106, 110). The adoptive transfer of mST2^+^ cells such as Tregs, IL-9-expressing T cells, and innate lymphoid cells showed the same effect. This is currently being evaluated in clinical trials (111, 112).

## Applications and Limitations of Biomarkers in Clinical Practice

Many specific and sensitive biomarkers for both aGVHD and cGVHD have been identified over the past decades. While these biomarkers can be exploited for patient-risk stratification, early GVHD assessment, monitoring of GVHD progression and for cost-effective management decision-making, no biomarkers are widely used in clinic. One of the main limitations for the application of biomarkers in clinics has been the lack of an adequate number of multicenter clinical trials. All candidate biomarkers require to be thoroughly validated from preclinical investigation to independent clinical research in large multicenter cohort setting(s) (9). In addition, it is important to minimize confounding variables or potential variables during studies by acquiring high-quality bio-samples that are selected, stored and processed rigorously. Last, collaboration between scientists and clinicians are encouraged to validate GVHD biomarkers from the bench for clinical use.

## Conclusion

Advances in technology in the field of omics have permitted the discovery of numerous biomarkers for identification of complications post-HCT and signature of the beneficial GVT. A good biomarker has several features: has been developed through the different phases including discovery and validation in large independent cohorts, use a cost efficient non-invasive robust assay that has been qualified. If, in addition the biomarker is mechanistic, the likelihood of this biomarker to be relevant is increased as for example ST2 that has been shown to be secreted by IFNγ producing T cells. If the biomarker is involved in the pathogenesis of the disease, it is likely that drugs (antibodies or small molecules) could target the pathway involved. Future directions should include aGVHD biomarkers preemptive trials. Biomarkers for other diseases such as autoimmunity should follow the same criteria.

## Author Contributions

DA and SP devised and wrote the manuscript. All authors contributed to critical revisions of the manuscript.

## Conflict of Interest

SP is an inventor on a patent on “Methods of detection of graft-versus-host disease” (US-13/573,766). KRC is on the advisory board of Jazz pharmaceuticals. CRYC is co-founder of Mana Therapeutics.

The remaining authors declare that the research was conducted in the absence of any commercial or financial relationships that could be construed as a potential conflict of interest.
